# SIN1 promotes the proliferation and migration of breast cancer cells by Akt activation

**DOI:** 10.1042/BSR20160192

**Published:** 2016-12-09

**Authors:** Deqiang Wang, Ping Wu, Hui Wang, Lei Zhu, Wei Zhao, Yuqin Lu

**Affiliations:** * Cancer Therapy Center, Affiliated Hospital of Jiangsu University, Zhenjiang, Jiangsu 212001, China; † Department of Pathology, Huai'an Maternity and Child Health Care Hospital, Huai'an, Jiangsu 223002, China; ‡ Jiangsu Jiankang Vocational College, Nanjing, Jiangsu 210029, China; § Department of Digestive System, Jiangsu Province Hospital of Traditional Chinese Medicine, Nanjing, Jiangsu 210029, China; ‖ Clinical Laboratory, Nanjing Maternity and Child Health Care Hospital, Nanjing, Jiangsu 210004, China; ¶ Department of Thyroid and Breast Surgery, The Second People's Hospital of Huai'an, Huai'an, Jiangsu 223002, China

**Keywords:** breast cancer, cell proliferation, human epidermal growth factor receptor 2 (HER2), migration, stress-activated protein kinase interacting protein 1 (SIN1), survival

## Abstract

Stress-activated protein kinase (SAPK) interacting protein 1 (SIN1) is an essential TORC2 component and a key regulator of Akt pathway that plays an important role in various pathological conditions including cancer. Whereas its functional role in breast cancer has not been well characterized. In the present study, SIN1 is associated with the progression and survival of breast cancer patients, as well as human breast cancer cell proliferation and migration. SIN1 mRNA level was significantly up-regulated in human breast cancer samples compared with their corresponding paracancerous histological normal tissues. Furthermore, the expression levels of SIN1 were also increased in three human breast cancer cell lines compared with human breast epithelial cell MCF10A. Overexpression of SIN1 promoted cell proliferation, colony formation and migration of breast cancer cells. Knockdown of SIN1 in MDA-MB-468 cells inhibited cell proliferation, colony formation and migration. In addition, SIN1 overexpression increased phosphorylation of Akt and knockdown of SIN1 inhibited phosphorylation of Akt in MDA-MB-468 cells. In a tumour xenograft model, overexpression of SIN1 promoted tumour growth of MDA-MB-468 cells *in vivo*, whereas SIN1 knockdown inhibits the tumour growth. Taken together, our results reveal that SIN1 plays an important role in breast cancer and SIN1 is a potential biomarker and a promising target in the treatment of breast cancer.

## INTRODUCTION

Breast cancer, a complex and intrinsically heterogeneous disease, is the leading type of cancer in women, accounting for 25% of all cases worldwide [1]. Survival rates are between 80% and 90% of those in England and the United States alive for at least 5 years [2]. However, in developing countries survival rates are poorer [1]. Oestrogen receptor (ER), progesterone receptor (PR) and human epidermal growth factor receptor 2 (HER2) are three important receptors on the surface or in the cytoplasm or nucleus of breast cancer cells [3]. Based on ER/PR, HER2 expression, breast cancer can be classified to four subtypes: ER/PR positive; HER2 positive; triple positive and triple negative [4]. For all of these, surgery and radiation are possible treatments, but ones that involve medications such as chemotherapy, hormone therapies and targeted therapies are different. They are specific to the subtype of cancer.

The mechanistic target of rapamycin (mTOR) is a highly conserved important regulator of cell growth and proliferation in all eukaryotes [5–7], and it functions as a critical and essential catalytic core in at least two known functionally distinct complexes, mTORC1 and mTORC2 [8,9]. Much is known about the functions and regulation of mTORC1, it's rapamycin sensitive and consists of mTOR, raptor and mLST8 (GβL) [9–12]. mTORC1 is involved in many cellular processes, including cell growth, nucleotide biosynthesis, lipogenesis, glycolysis and autophagy [13]. mTORC2 is rapamycin-insensitive and composed of mTOR, rictor (mAVO3), mLST8 (GβL) and stress-activated protein kinase (SAPK) interacting protein 1 (SIN1) [14,15]. mTORC2 controls growth by regulating lipogenesis, glucose metabolism, the actin cytoskeleton and apoptosis [13]. Phosphorylation of SIN1 at Thr^86^ and Thr^398^ suppresses mTORC2 kinase activity by dissociating SIN1 from mTORC2, and inhibits downstream Akt signalling to suppress tumorigenesis [16]. SIN1 plays an important role in hepatocellular carcinoma invasion and metastasis by facilitating epithelial–mesenchymal transition [17]. In the present study, we investigated the potential functions of SIN1 in human breast cancer.

## MATERIALS AND METHODS

### Patients and samples

The present study was approved by the Ethics Committee of The Second People's Hospital of Huai'an, Huai'an, Jiangsu, China. All patients provided their full consent to participate in the present study. The patients enrolled in the present study underwent curative surgery without prior treatments. Tissue specimens were examined separately by two pathologists under double-blinded conditions without prior knowledge of the clinical status of the specimens. The patients’ medical records were reviewed to obtain data including age at diagnosis, sex, tumour location, tumour size (diameter), lymph node metastasis, histology, tumour invasion and TNM stage according to the guidelines of American Joint Committee. Detailed clinical histopathological factors were presented in Table 1. For the measurement of prognosis, we analysed the clinical data overall survival (OS), defined as the time from surgery to death. All recruited patients had been followed-up periodically until the due date.

**Table 1:** Association between SIN1 expression and clinicopathological features of breast cancer *P<0.05 is considered significant.

	Patients	SIN1 expression in breast cancer tissues			
Varials	number (*n*)	Low	High	χ^2^	*P*
**Age (Years)**				0.151	0.698
≤50	36	14	22		
>50	44	19	25		
**Menstruation status**				0.755	0.385
Premenopause	39	18	21		
Postmenopause	41	15	26		
**Tumour size (cm)**					
≤2	27	17	10	7.928	0.005*
>2	53	16	37		
**Histological grade**				9.699	0.008*
I	17	11	6		
II	43	19	24		
III	20	3	17		
**Lymph nodes status**				5.224	0.022*
Negative	34	19	15		
Positive	46	14	32		
**TNM grade**				8.299	0.016*
I	10	6	4		
II	28	16	12		
III–IV	42	11	31		
**ER**				0.172	0.679
−	27	12	15		
+	53	21	32		
**PR**				0.860	0.354
−	29	10	19		
+	51	23	28		
**HER2**				0.309	0.578
−	32	12	20		
+	48	21	27		

### Immunohistochemistry analysis

Immunohistochemistry (IHC) was performed using the avidin–biotin peroxidase complex method with all breast carcinoma samples. All sections were deparaffinized in xylene and dehydrated through a gradient concentration of alcohol before endogenous peroxidase activity was blocked using 0.5% H_2_O_2_ in methanol for 10 min. After non-specific binding was blocked, the slides were incubated with mouse monoclonal antibodies for SIN1 (1:100; Cell Signaling Technology) in PBS at 4°C overnight in a humidified container respectively. Next day, biotinylated goat anti-rabbit IgG (1:400; Sigma–Aldrich) was incubated with the sections for 1 h at room temperature and detected using a streptavidin–peroxidase complex. The brown colour indicative of peroxidase activity was developed by incubation with 0.1% 3,3′-diaminobenzidine (Sigma–Aldrich) in PBS with 0.05% H_2_O_2_ for 5 min at room temperature. Polyclonal HER2 antibody in the Herceptin kit (HercepTest, DAKO) was used according to the manufacturer's instructions. Positive controls of known positive breast cancer tissues and negative controls with primary antibody replaced with TBS were run with the patient slides in each run of IHC.

### Cell culture and lentiviral transduction

HEK293T and human breast cancer cells MCF7 were cultured in DMEM containing 10% FBS. Human normal breast epithelial cell lines MCF10A were cultured in DMEM/F12 medium supplemented with 10% FBS. Human breast cancer cells SKBR3 were cultured in RPMI 1640 and MDA-MB-468 were cultured in L15containing 10% FBS. All media and FBS were from Invitrogen. For lentiviral transduction, HEK293T cells (7×10^6^) were plated in a 15 cm dish, incubated for 24 h and then transfected with 15 μg of lentivirus plasmids. After 48 h, the virus containing medium was filtered through a 0.45 μm filter (Millipore) and collected as first supernatant. Additional medium with FBS was added into the plate and the virus-containing medium was filtered and collected as second supernatant after 24 h. Both supernatants were centrifuged at 20000 ***g*** for 2 h. The supernatant was abandoned and the precipitate was suspended in 100 μl DMEM. For infections, gradient virus-contained DMEM was added into the medium with polybrene (Sigma–Aldrich, 4 μg/ml), and then the culture plates were incubated at 37°C for 6 h and replaced by full medium. After incubation for 36–48 h, the infected cell populations were confirmed by fluorescence microscope for GFP expression to evaluate the virus titre. Target cells were plated in six-well plates for infections by appropriate virus-contained DMEM.

### MTT and colony formation assays

For MTT assay, the cells were seeded at a density of 5×10^3^ cells/well into a sterile 96-well plate and grew for 24, 48 and 72 h. Cell viability was measured by MTT assay from Sigma–Aldrich. Twenty microlitres of 5 mg/ml MTT were added to each well and incubated with cells for 4 h in the incubator. The formazan was dissolved in 100 μl of DMSO followed by removal of the medium. Finally, the absorbance was measured using a spectrophotometer at an absorption wavelength of 570 nm.

For colony formation assay, the cells were seeded in to six-well plate with 400 cells per well. Approximately 5 days later, the clones were washed with 1× PBS and stained with Crystal Violet for approximately 20 min. Finally, the clones were imaged and quantified.

### Antibodies

The antibodies purchased were as follows: anti-Akt and anti-pAkt from Santa Cruz Biotechnology; anti-SIN1 and anti-β-actin from Cell Signaling Technology.

### Cell migration assays

For transwell assay, the cells were seeded into the upper transwell chamber (8-μm pore size, Transwell, Corning) without FBS, and medium containing 10% (v/v) FBS in the lower chamber served as attractant. After incubating for 24 h, cells that migrated to the underside of the membrane were fixed and stained with Crystal Violet. Images were taken under a microscope (Olympus).

### Nude mice xenograft model

Four MDA-MB-468 cell lines were constructed, including SIN1 overexpression cell, SIN1 knockdown cell and control cells for both SIN1 overexpression and knockdown respectively. The cells in the exponential phase of growth were trypsinized, rinsed with 1× PBS for three times, and then resuspended in 100 μl of 1× PBS. Each six nude mice (6 weeks old, male) was inoculated subcutaneously with a clonal population of MDA-MB-468 cell (5×10^6^ cells). Xenograft tumour sizes were measured by measuring two perpendicular diameters with digital calibres every 1 week after appearance of tumours and calculated by the formula 0.5 × length × width^2^. All of the mice were killed 5 weeks after inoculation and the tumours were removed instantly.

### Statistical analyses

Data were presented as the means ± S.D. from three independent experiments. Two-tailed Student's *t* tests were used to evaluate the data. The differences between groups of SIN1 expression in breast cancer tissues were analysed using the Chi-squared test (χ^2^ test). The log-rank test was used to explore the associations between SIN1 expression and the OS of breast cancer patients. All of the statistical analyses were performed with SPSS 19.0. The difference was considered to be statistically significant at *P<0.05 and **P<0.01.

## RESULTS

### SIN1 is up-regulated in primary breast cancer tissues

**Figure 1:**
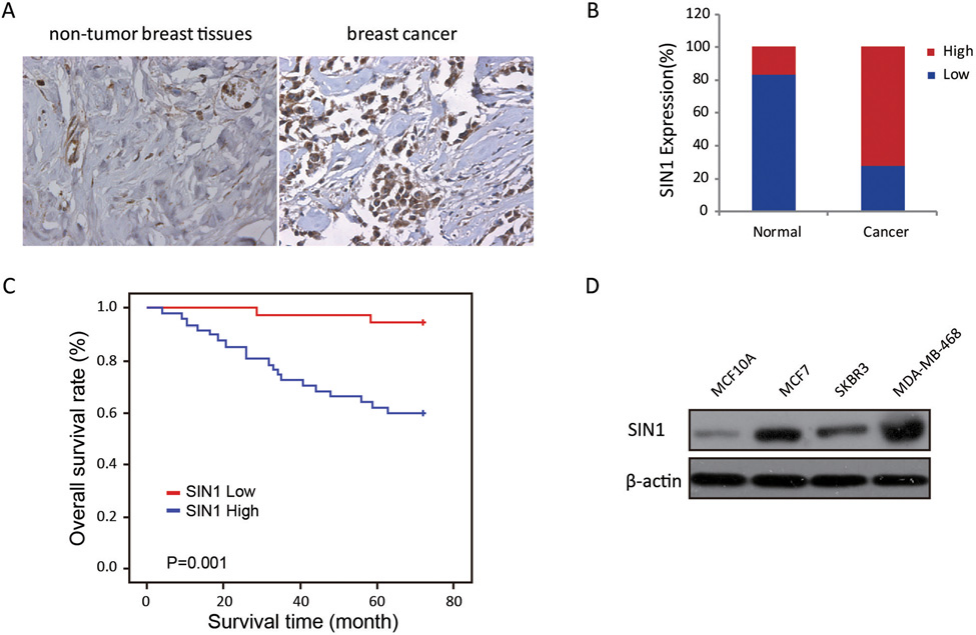
SIN1 expression was elevated in breast cancer tissues and cells (**A**) The expression of SIN1 was detected in breast normal tissue and cancer tissue by IHC, and representative samples are shown at 400× magnification. (**B**) The expression of SIN1 was quantified by H-score in breast normal tissue and cancer tissue. The samples were classified as low (H-score<200) or high (H-score≥200) SIN1 expression. (**C**) The OS rates of the 80 breast cancer patients were compared with the SIN1 Low and SIN1 High groups. Statistical significance was determined using the log-rank test. (**D**) The expression level of SIN1 in the three breast cancer cells were detected by Western blot.

To detect the expression of SIN1 in breast cancer, we examined SIN1 expression in 80 breast cancer tissues and 30 normal tissues by IHC. SIN1 was obviously up-regulated in breast cancer tissues compared with normal tissues (Figure 1A). Most of the breast cancer tissues (72.5%, 58/80 cases) were found to exhibit high SIN1 expression. In contrast, most of the normal tissues (83.3%, 25/30 cases) expressed low SIN1 (Figure 1B).

We next investigated the association of SIN1 expression level with the survival of the patients. The log-rank test revealed that SIN1 expression correlated significantly with the OS of breast cancer patients (Figure 1C). The OS for breast cancer patients with high SIN1 expression was significantly shorter than those patients with low SIN1 expression (*P*=0.001).

Upon clinicopathological correlation analysis, elevated SIN1 protein levels are positively correlated with tumour size, histological grade, lymph nodes status and TNM grade advanced tumour stage of breast cancer (Table 1).

### SIN1 is up-regulated in human breast cancer cells

To study the role of SIN1 in breast cancer, we used one normal breast epithelial cell line MCF10A and three different breast cancer cell lines. MCF7 is ER^+^, PR^+/−−^ and HER2^−−^. MDA-MB-468 cell line is triple-negative. SKBR3 cell line is ER^−−^, PR^−−^ and HER2^+^. We analysed the SIN1 expression levels of these cell lines. We found that MDA-MB-468 cells had the highest level of SIN1 expression, much higher than MCF7 and SKBR3 (Figure 1D).

### SIN1 overexpression promotes cell proliferation of breast cancer cell lines

**Figure 2:**
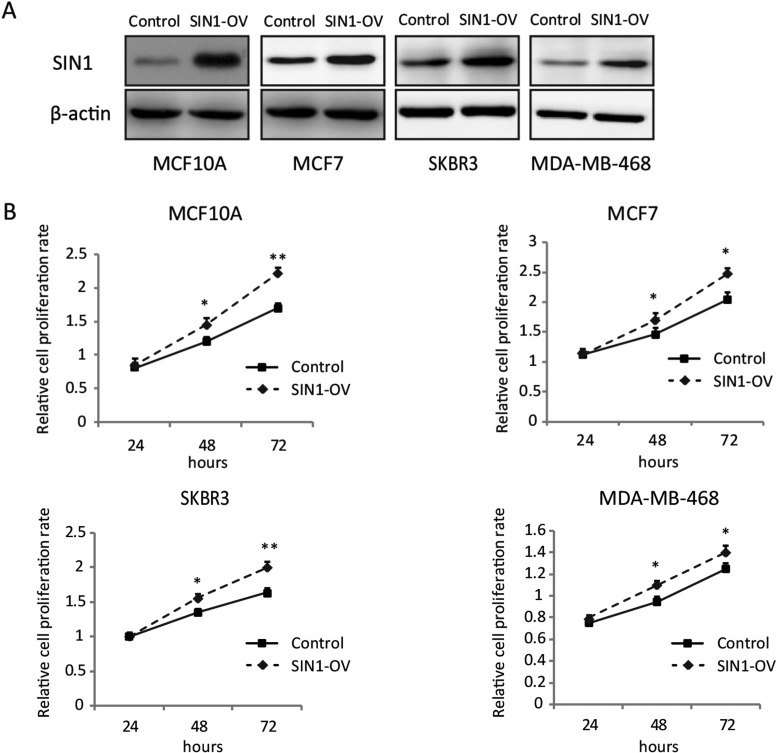
SIN1 overexpression promoted human normal breast epithelial cell MCF10A and breast cancer cells proliferation (**A**) The expression levels of SIN1 in control and SIN1-overexpressing cells by lentivirus infection were detected by Western blot. (**B**) MTT assay was used to determine the cell proliferation rate of the cells with or without overexpression of SIN1 at the time point as indicated. The data are shown as mean ± S.D.; *P<0.05 and **P<0.01.

We also established MCF10A and these three breast cancer cell lines with stable expression of SIN1 by lentivirus infection. Western blot analysis was conducted to determine the expression level of SIN1 in these cells (Figure 2A). The cell proliferation rate of these cells was investigated by MTT assay. The cell growth rates of all these four cell lines were significantly increased by SIN1 overexpression (Figure 2B).

### SIN1 promotes colony formation and migration of breast cancer cells

**Figure 3:**
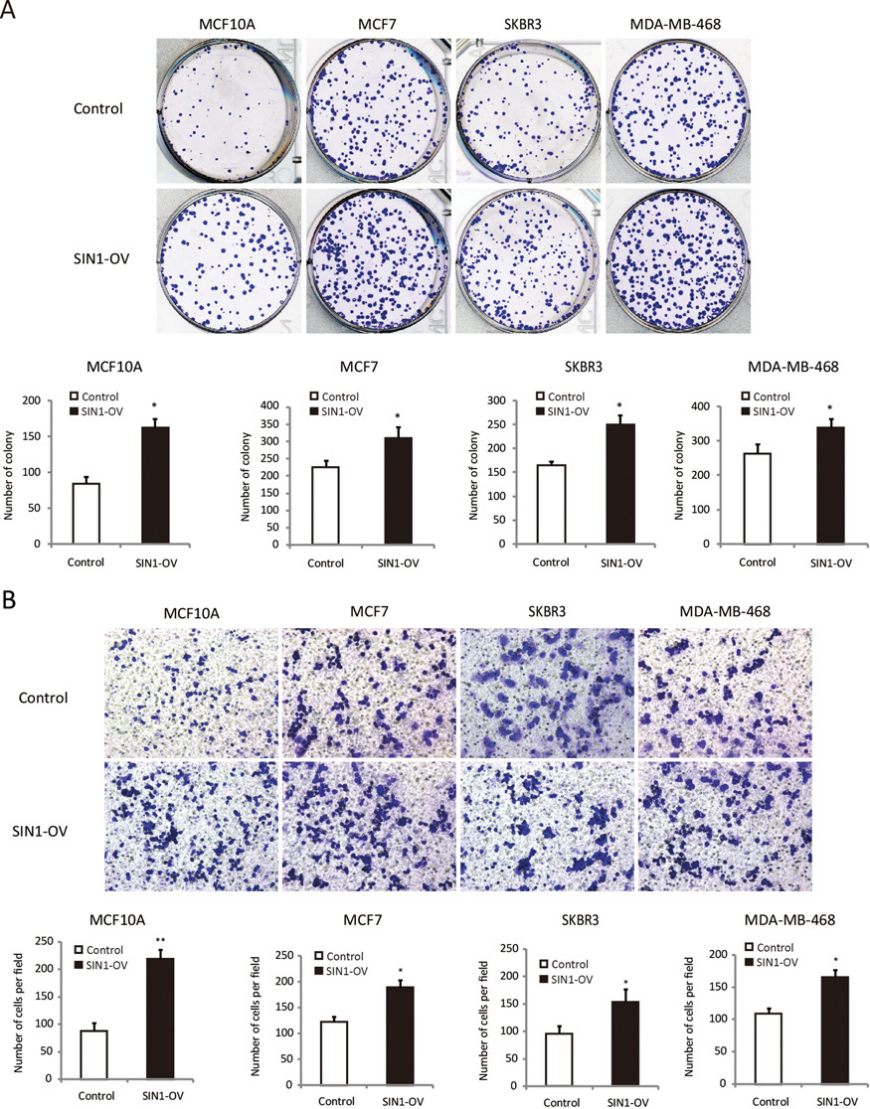
SIN1 overexpression promoted colony formation and migration of breast epithelial cell and breast cancer cells (**A**) Effect of SIN1 overexpression on colony formation was measured in MCF7, SKBR3 and MDA-MB-468 cells. The cells were seeded into six-well plates and cultured for 5 days, followed by Crystal Violet staining. The colony counts were shown below the graph. (**B**) A transwell assay was performed with the cells to measure the migration rate. Statistical results of the data means ± S.D. are shown in the below panel. Three independent experiments yielded similar results. Statistical significance was calculated using the Student's *t* tests when only two groups were compared; *P<0.05 and **P<0.01.

Next, colony formation was used to investigate the potential of tumorigenesis of these cells. We found that SIN1 overexpression profoundly promoted colony formation in these cells (Figure 3A). Therefore, these data suggest that SIN1 promotes the growth of breast cancer cells.

We also analysed the potential role of SIN1 on the migratory activity of breast cancer cells. The transwell assay revealed that the cell migration rate was significantly increased by SIN1 overexpression in these breast cancer cells (Figure 3B). These data indicate that in addition to the regulatory function on cell proliferation, SIN1 has an impact on the migration of breast cancer cells.

### Knockdown of SIN1 in MDA-MB-468 cells inhibits cell proliferation and migration

**Figure 4:**
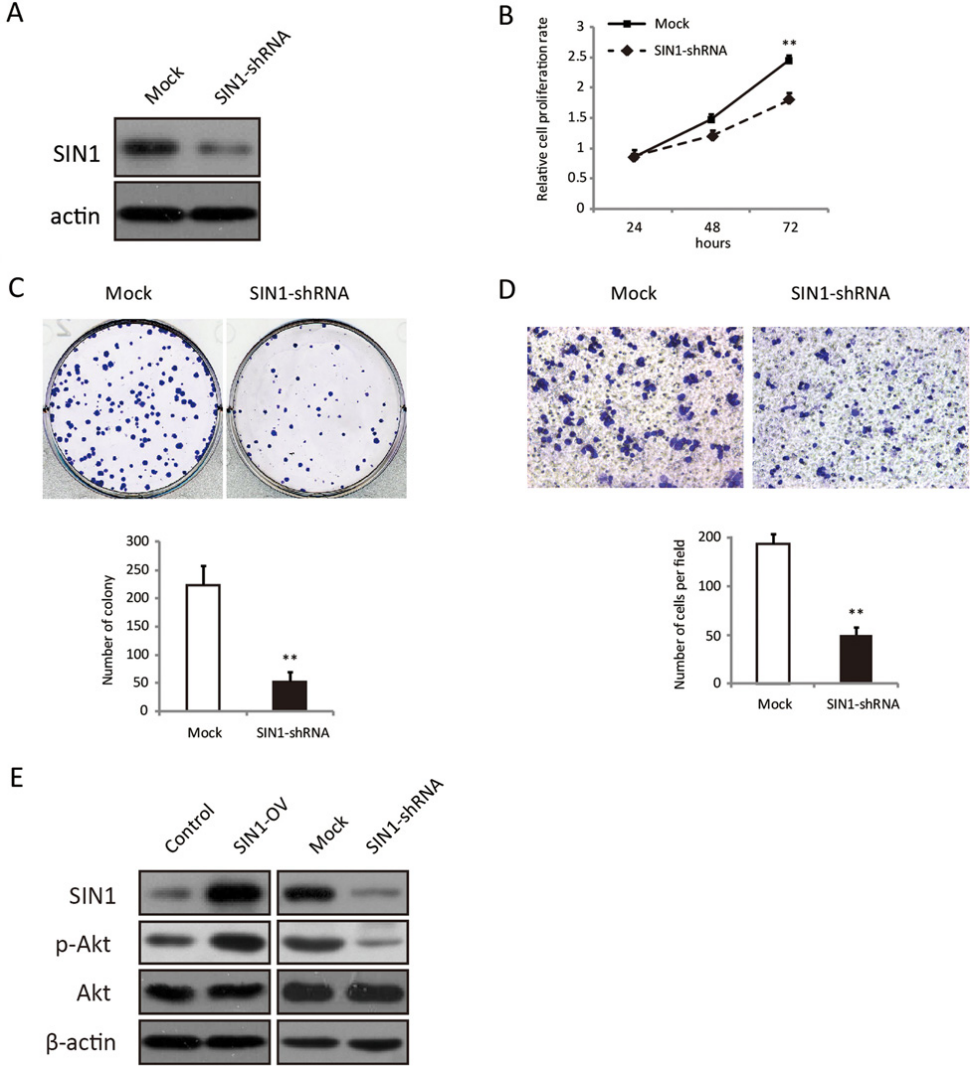
Knockdown of SIN1 in MDA-MB-468 cells decreased cell growth and migration (**A**) The expression level of SIN1 in MDA-MB-468 cells infected with lentivirus containing mock or SIN1-specific shRNA was detected by Western blot. (**B**) Cell proliferation rate of these cells was determined by MTT assay at the indicated time point. (**C**) Colony formation assay was performed in MDA-MB-468 cells that were stained by Crystal Violet. The statistical results are shown in the lower panel. (**D**) Transwell assay was performed with MDA-MB-468 cells, followed by photography and counting. (**E**) Western blot analysis was employed to detect the expression level of p-Akt, Akt with SIN1 overexpression and knockdown in MDA-MB-468 cells. The statistical results are shown in the lower panel. All the data are shown as mean ± S.D.; **P<0.01.

As shown in Figure 1(D), MDA-MB-468 cell line had the highest expression level of SIN1 among the three breast cancer cell lines. We further analysed the function of SIN1 by establishing a MDA-MB-468 cell line with down-regulation of SIN1 by lentivirus infection. A control shRNA or SIN1-specific shRNA was introduced into MDA-MB-468 cells. As confirmed by Western blot analysis, SIN1 protein was successfully reduced by a SIN1-specific shRNA (Figure 4A). Knockdown of SIN1 could decrease cell proliferation rate of MDA-MB-468 cells by MTT assay (Figure 4B). The capacity of colony formation was also reduced by SIN1 knockdown (Figure 4C). Consistently, the migration ability of MDA-MB-468 cells was significantly impaired by SIN1 down-regulation by the transwell assay (Figure 4D). These data, therefore, further confirmed that SIN1 promotes the proliferation and migration of human breast cancer cells.

### SIN1 promotes Akt activation in MDA-MB-468 cells

Dysregulation of the phosphoinositide 3-kinase (PI3K)/Akt pathway is involved in tumorigenesis by regulating cell proliferation, migration and invasion [18,19]. SIN1 is considered as a critical regulator in the Akt pathway by controlling Akt-Ser^473^ phosphorylation and Akt activation [17]. To investigate the molecular mechanism for SIN1-regulated cell proliferation and migration, MDA-MB-468 cells were infected with lentivirus expressing SIN1, SIN1-shRNA and their control lentivirus respectively. And we assessed the activation of Akt in MDA-MB-468. The results showed that phosphorylation levels of Akt were increased with SIN1 overexpression and pAkt was reduced by SIN1 knockdown (Figure 4E).

### SIN1 promotes the growth of the breast cancer *in vivo*

**Figure 5:**
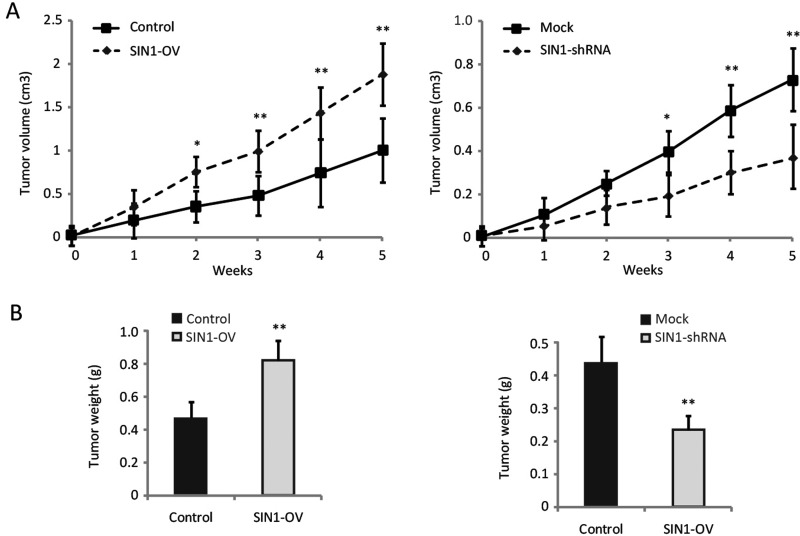
SIN1 promoted the growth of the breast cancer *in vivo* (**A**) Tumour volume was calculated every 7 days after the injection of MDA-MB-468 cells stably overexpressing SIN1 or with SIN1 knockdown. Tumour volume was calculated according to the formula 0.5 × length × width^2^. (**B**) The weight of the tumours. The statistical results are shown in the lower panel. All the data are shown as mean ± S.D.; *P<0.05 and ** *P*<0.01.

Next, we used a xenograft model to further elucidate the effect of SIN1 in tumour growth of breast cancer *in vivo*. MDA-MB-468 cells with stable expression of either SIN1 or SIN1 knockdown were implanted into the nude mice. These mice were killed in 5 weeks and the tumours were removed. The growth of the breast cancer cells in the mice was measured by tumour volume and tumour weight. Tumour weight was significantly increased by SIN1 overexpression and decreased by SIN1 deregulation (Figure 5A). And tumour weight was also increased by SIN1 overexpression and reduced by SIN1 knockdown in these mice (Figure 5B). These observations, therefore, clearly indicated that SIN1 has a powerful activity to suppress tumorigenicity of breast cancer *in vivo*.

## DISCUSSION

SIN1 is essential for early embryonic development and is a key regulator of Akt, which plays an important role in cancer [20,21]. SIN1 promotes HCC invasion and metastasis by facilitating epithelial–mesenchymal transition [17]. However, it is unclear whether SIN1 plays a role in the development of human breast cancer.

In the present study, we have provided convincing evidence that SIN1 functions as a tumour promoter in human breast cancers. In the clinical breast cancer samples, SIN1 expression level was robustly up-regulated as compared with the adjacent normal tissues. Furthermore, SIN1 expression status was associated with the survival of the breast cancer patients. SIN1 overexpression promoted cell proliferation, colony formation and migration of human breast cancer cells. Knockdown of SIN1 in MDA-MB-468 cells significantly inhibited cell growth and migration both *in vitro* and *in vivo*. Mechanistically, SIN1 is able to activate Akt signalling pathway by promoting the phosphorylation of Akt in MDA-MB-468 cells and such activation likely underlies its oncogenetic activity in these cells.

Taken together, our study shows, for the first time, that SIN1 is overexpressed in human breast cancer and its overexpression is significantly correlated with a poor prognosis of breast cancer. Mechanistically, we have demonstrated that SIN1 promotes breast cancer cell proliferation and migration both *in vitro* and *in vivo* by up-regulating phosphorylation of Akt. High expression levels of SIN1 may serve as a novel molecular marker for human breast cancer and a promising target for drug development.

## Abbreviations

ER: oestrogen receptor
HER2: human epidermal growth factor receptor 2
IHC: immunohistochemistry
mTOR: mechanistic target of rapamycin
OS: overall survival
PR: progesterone receptor
SAPK: stress-activated protein kinase
SIN1: SAPK interacting protein 1
